# Risk of Cancer Recurrence Exerts the Strongest Influence on Choice Between Active Surveillance and Thyroid Surgery as Initial Treatment for Low‐Risk Thyroid Cancer: Results of a Discrete Choice Experiment

**DOI:** 10.1002/wjs.12520

**Published:** 2025-03-05

**Authors:** Jacob Hampton, Gavin Cooper, Laura Wall, Christopher Rowe, Nicholas Zdenkowski, Elizabeth Fradgley, Julie Miller, Jenny Gough, Scott Brown, Christine O'Neill

**Affiliations:** ^1^ Surgical Services John Hunter Hospital Newcastle Australia; ^2^ School of Medicine and Public Health University of Newcastle Newcastle Australia; ^3^ Hunter Medical Research Institute Newcastle Australia; ^4^ School of Psychological Sciences University of Newcastle Newcastle Australia; ^5^ Department of Endocrinology John Hunter Hospital Newcastle Australia; ^6^ Department of Surgery The Royal Melbourne Hospital Melbourne Australia; ^7^ Department of Surgery Wesley Hospital Brisbane Australia

**Keywords:** active surveillance, discrete choice experiment, thyroid cancer, thyroid surgery

## Abstract

**Background:**

Treatment options for low‐risk differentiated thyroid cancer (DTC) include active surveillance (AS), hemithyroidectomy (HT), or total thyroidectomy (TT). Improved understanding of patient values and preferences is required to inform shared decision‐making. This study examined factors influencing patient treatment preferences and trade‐offs for low‐risk DTC.

**Methods:**

Adult participants with benign thyroid nodules or low‐risk DTC completed an online discrete choice experiment (DCE). Utilizing the scenario of a 50‐year‐old person with a small solitary DTC, participants chose between three unlabeled treatment options (representing AS, HT, and TT). Risk profiles varied across 5 domains: voice change, thyroid hormone supplementation, hypocalcaemia, chance of future thyroid surgery, and 10‐year risk of cancer recurrence. Participants self‐reported demographics, disease factors, and answered a decisional regret scale. A conditional logit model was utilized.

**Results:**

The DCE was completed by 143 patients across three sites. The conditional logit model demonstrated that participants preferred AS (49%) over TT (29%) or HT (22%). All five domains influenced choices (all *p* < 0.001), but perceived risk of cancer recurrence exerted most influence. Cancer survivors chose AS less often than those with benign disease (46% vs. 57%), driven by perceived risks of further surgery and cancer recurrence. As the perceived risk of cancer recurrence increased, more participants preferred HT over AS.

**Conclusion:**

This study demonstrates that when blinded to the actual treatment, patients prefer the trade‐offs associated with AS rather than TT or HT. Perceived risk of cancer recurrence exerted the greatest influence. Accurate risk stratification for cancer recurrence is critical to shared decision‐making.

## Graphical abstract

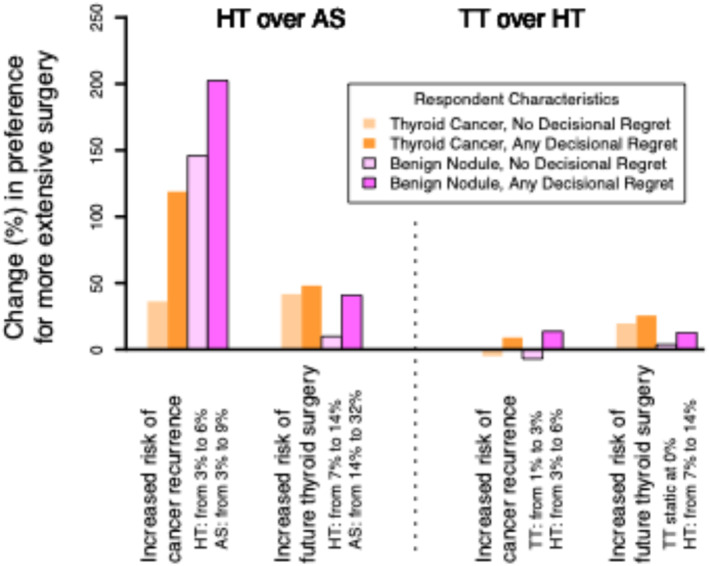

## 1 Introduction

The incidence of differentiated thyroid cancer (DTC) has increased significantly in recent decades in part due to increased imaging and incidental findings [1, 2, 3, 4]. The global survival rate for thyroid cancer is excellent, greater than 98% at 5 years [5] In patients diagnosed with DTC, the initial surgical treatment choice may not improve mortality [6, 7, 8]. To minimize harm from overtreatment, the 2015 American Thyroid Association (ATA) Guidelines promote active surveillance (AS) or hemithyroidectomy (HT) instead of total thyroidectomy (TT) as treatment options for patients with small low‐risk DTC [9]. However, translation of the paradigm of treatment de‐escalation has been slow, particularly with respect to AS, with barriers to implementation exhibited by both patients and clinicians [10, 11]. Facilitation of greater patient involvement in treatment decisions may influence how often AS is used [12].

Shared decision‐making (SDM) is an important care paradigm between patients and clinicians that facilitates collaboration and agreement about treatment by building consensus and sharing information [13, 14, 15]. SDM is recommended in Australia and internationally as preferred practice to engage and empower patients [16, 17]. The ATA encourages SDM to help clinicians better understand the concerns and preferences of patients and therefore optimize care pathways. Other benefits of SDM include improved health‐related quality of life, reduced anxiety and regret, increased trust in the healthcare system, and avoidance of unnecessary treatment [14, 16, 17]. Clinicians face barriers implementing SDM [15] including a lack of informational resources, lack of training in SDM techniques, and lack of time. To support implementation of SDM, discrete choice experiments (DCEs) can be used to quantify patients' health preferences and values around treatment decisions [18].

Four prior DCEs have investigated patient preferences regarding initial management of thyroid cancer [19, 20, 21, 22]. All have identified decisional trade‐offs between cancer recurrence, surgical complications, and thyroid hormone supplementation requirement. To date, no DCE has presented patients with a choice between three options for low‐risk DTC, namely: AS, HT, or TT. Therefore, to support implementation of SDM and address the expanded treatment options for low‐risk DTC, we conducted a prospective cross‐sectional cohort DCE to quantify the trade‐offs that patients are willing to make between the three described management options for low‐risk DTC and to map the contribution of prior health experiences (via decisional regret).

## 2 Material and Methods

### 2.1 Patient Population

Participants were included if they were > 18 years with a diagnosis of a benign thyroid nodule or low‐risk DTC (as per the ATA guidelines) [9]. They were also required to have already undergone surgery or not recommended to undergo surgery for their thyroid nodule. Participants were excluded if they had intermediate or high‐risk DTC (as per the ATA guidelines); were awaiting a thyroid procedure; and had insufficient English skills or had a physical or psychological condition that would preclude questionnaire completion. Two recruitment methods were used: postcards and phone calls. Postcards (containing study information and a quick response (QR) code, Supplementary Information S1) were distributed by clinicians following a consultation (often the first postoperative appointment). Participants for phone recruitment had confirmed low‐risk DTC and had indicated willingness to participate in thyroid cancer research after prior studies [12, 23].

### 2.2 DCE Development and Construction

**FIGURE 1:**
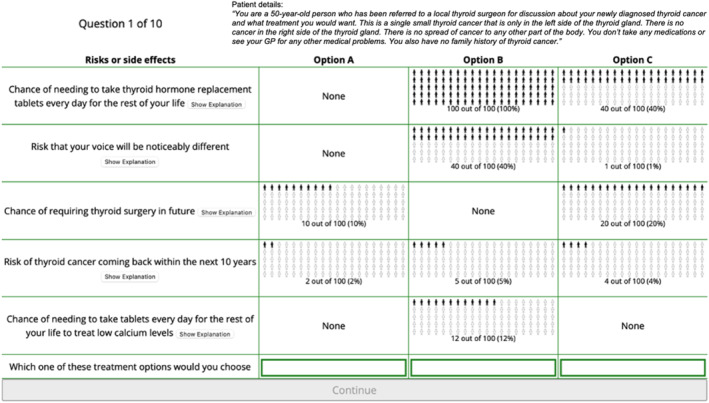
Example choice set from the DCE. Screenshot of one example choice set from the DCE, presenting the static clinical vignette (top); the five risk attributes (left column) which were static for each question, but which differed in order across participants; and the three unlabeled treatment choices (option A, B, and C, representing AS, TT, and HT presented in random order for each question) and the risk levels for each attribute and choice (presented graphically and as text, with risk levels varying for each of the 10 questions within the ranges specified in Table 1).

The DCE was conducted following published standards [24]. Patients were presented with the clinical scenario of a 50‐year‐old with a solitary left sided DTC, euthyroid, with no comorbidities, and no prior personal or family history of thyroid cancer. Ten sets of questions asked participants to choose from three unlabeled treatment options (order randomized), with varying risks across 5‐domains including voice change, thyroid hormone supplementation, hypocalcaemia, chance of future thyroid surgery, and 10‐year risk of cancer progression (Figure 1/Supplementry Information S2). Pictographs were used to better communicate the probabilistic information [19, 21, 25, 26]. Levels and risk attributes used in this DCE are presented in Table 1 and were based on the literature review [27, 28, 29, 30, 31, 32]. A “patient‐centric” approach was taken to the definition of these attributes, such that “the risk that your voice will be notably different” reflects more than just the risk of recurrent laryngeal nerve palsy and that “the need to take calcium supplements” is more expansive than those with undetectable PTH levels.

**TABLE 1:** DCE risk attributes and risk levels.

	Risk levels (patients were blinded to the actual treatment choice and were presented three unlabeled treatment choices)		
Risk attributes	TT	HT	AS
Risk that your voice will be noticeably different	2%–40% (10%)	1%–20% (10%)	0% (0%)
Chance of needing to take thyroid hormone replacement tablets every day for the rest of your life	100%	20%–60% (40%)	0% (0%)
Chance of needing to take tablets every day for the rest of your life to treat low calcium levels	1%–12% (5%)	0% (0%)	0% (0%)
Chance of requiring thyroid surgery in future	0%	5%–20% (10%)	10%–50% (20%)
Risk of thyroid cancer coming back within the next 10 years	1%–5% (2%)	2%–10% (4%)	2%–15% (5%)

*Note:* The percentage range presents the maximal clinically plausible variation for each treatment choice that could be presented to participants as part of the DCE. The number in brackets represents a likely typical risk level for the average patient, consistent with published evidence, and was used in the base MNL model.

Abbreviations: AS, active surveillance; HT, hemithyroidectomy; and TT, total thyroidectomy.

**FIGURE 2:**
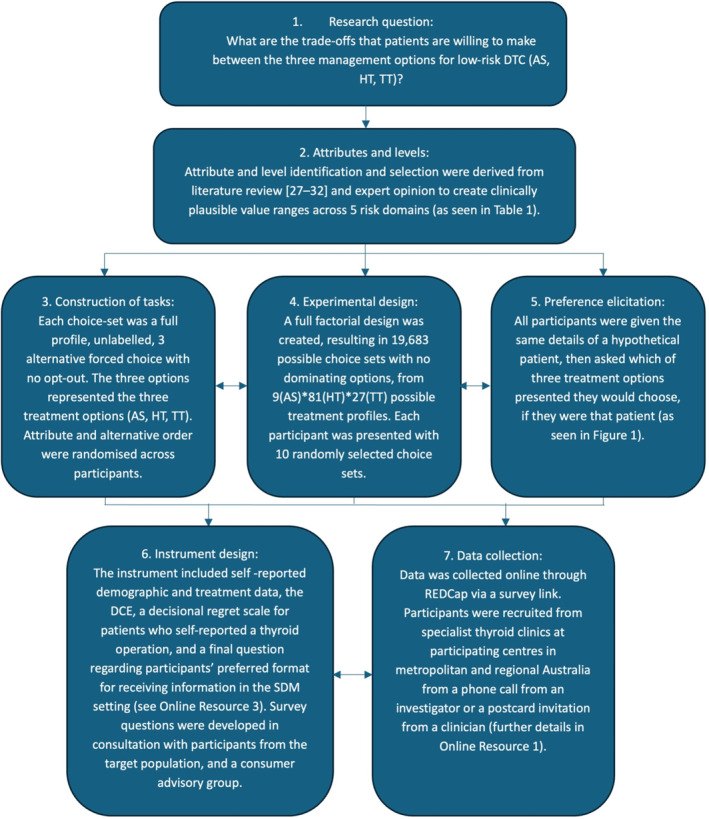
DCE design schema. The first 7 stages of the Conjoint Analysis Applications in Health Checklist from the ISPOR Good Research Practices for Conjoint Analysis Task Force [24] with details for this study. The final 3 stages of the checklist (8. statistical analyses, 9. results and conclusions, and 10. study presentation) are described and presented in the results and discussion sections of the paper. AS, active surveillance; DCE, discrete choice experiment; DTC, differentiated thyroid cancer; HT, hemithyroidectomy; REDCap, Research Electronic Data Capture; SDM, shared decision making; and TT, total thyroidectomy.

The details of the DCE process are summarized in Figure 2. Additional survey questions assessed decisional regret (validated 5‐item, 5‐point Likert scale) which were dichotomized as present/absent and then incorporated into the MNL model as a potential influencing factor [33, 34]. These were administered to patients who had undergone prior thyroid surgery to assess their regret and how this may influence their DCE responses. Additional information about decisional regret and information needs beyond the consultation are contained in Supplementry Information S3.

The study was approved by the Hunter New England Human Research Ethics Committee (2021/ETH10849) and participants provided informed consent. The survey was developed with consumer input and piloted by 20 participants. No changes were required, and hence, these participants were included in the final analysis.

### 2.3 DCE Analysis

The discrete choice responses were analyzed using a conditional logit model (CLM), a type of random utility model commonly used for DCEs [35, 36]. The model assumes that each treatment option leads to an internal “utility” and choices are made by selecting the option with the largest utility. The utility for each option fluctuates randomly around an average value, which is determined by the attributes of that option. We estimated how each attribute contributes to the average utility using the clogit function from the “survival” package in the programming language R [37, 38].

## 3 Results

The DCE was completed by 143 participants across three Australian sites, Table 2. The decisional regret scale was completed by 132 postoperative participants with 36 (27%) expressing mild regret (score 1–24/100) and 17 (13%) moderate to severe regret (25–100/100) (Supplementry Information S4). Additional information (beyond the consultation with a doctor) was desired by 64% of respondents with 29% preferring online information, 23% paper, and 48% both online and paper.

**TABLE 2:** Participant characteristics, decision regret, and information needs.

	*N* = 143 (%)
Age (years), mean (SD)	56.3 (SD 13.6)
Sex	
Female	108 (76%)
Male	35 (24%)
Recruitment^a^	
Phone call	49 (34%)
Postcard	94 (66%)
Disease type	
Low‐risk differentiated thyroid cancer	57 (40%)
Benign thyroid nodule	86 (60%)
Postoperative participant self‐reported health change	137 (96%)
New calcium supplementation	12/114^b^ (11%)
New voice change following surgery	34/114^b^ (30%)
New thyroid hormone replacement	55/114^b^ (48%)
Decision regret^c^	*N* = 132 (%)
None	79 (60%)
Mild	36 (27%)
Severe	17 (13%)
Postoperative time (months), median (range)	19 (2–288)
Information desired beyond the consultation	*N* = 140 (%)
Participants desiring additional information	89 (64%)
Online resource preferred	26 (29%)
Hard copy resource preferred	20 (23%)
Both online and hard copy resource preferred	43 (48%)

*Note:* 132 responded to the decision regret scale and 140 indicated their additional information needs.

^a^ 49 of 89 (55%) eligible patients contacted by phone completed surveys. No denominator can be calculated for postcard recruitment.

^b^ Of the 137 postoperative patients, 114 participants responded to the questions regarding calcium supplementation, voice change, and thyroid hormone replacement.

^c^ Additional data relating to decisional regret are contained in Supplementary Information S4.

### 3.1 Conditional Logit Model (CLM)

All five attributes had statistically significant influences on choices (all *p* < 0.001), but the perceived risk of cancer recurrence exerted most influence (*β* = −9.9 (SE = 0.68) compared with *β* > −2.8 for all other factors) (Supplementry Information S5). Using a CLM to predict choice proportions, when the typical risk levels for each attribute were applied, participants preferred the perceived risks and experiences associated with AS (49%), with fewer preferring TT (29%) or HT (22%). This means, that for the typical risk scenario, there is essentially an equivalence of choice between surgery and AS.

On average, participants with a history of DTC chose AS less often than those with benign thyroid disease (46% vs. 57%), driven by the attributes related to perceived risk of cancer recurrence or needing more than one operation. Respondents with a history of neck surgery who reported any degree of decisional regret chose the blinded option representing AS more often than surgery (65% vs. 43%), with differences primarily driven by the perceived risk of needing further surgery and perceived risk of voice change.

### 3.2 Consequences of Variation in Risks

The predicted choices from the CLM were used to explore the effect of varying each risk within a clinically plausible range for the same hypothetical clinical scenario. We report these results using focused choices between AS and HT and between HT and TT.

**FIGURE 3:**
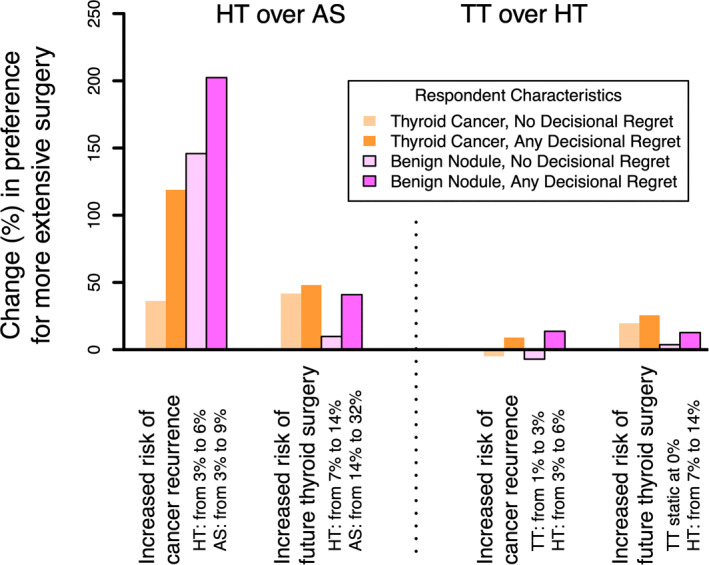
Choice preferences between HT and AS (panel A) and between TT and HT (panel B) following variation in perceived risks that are intrinsic to the cancer. For example, in Panel A, as the risk of cancer recurrence increases from 3% to 6% following HT and from 3% to 9% following AS, participants are more likely to choose HT over AS, with greatest change (∼200%) for participants with decisional regret and without a personal history of thyroid cancer. AS, active surveillance; HT, hemithyroidectomy; and TT, total thyroidectomy.

The effect of perceived cancer‐associated risks on treatment choice is presented in Figure 3. As the perceived risk of cancer recurrence increased, more participants preferred HT over AS. The greatest effect size was seen in this group with a 200% increase in preference for more extensive surgery in participants with decisional regret but without thyroid cancer (Figure 3). In contrast, an increased perceived risk of cancer recurrence had minimal effect on preference of TT over HT. Alternatively, this can be described as what perceived risk of cancer recurrence threshold a patient would consider more extensive surgery over AS. When the perceived risk of recurrence is almost double the clinical standard risk, the choice for TT exceeds the choice for AS (Supplementry Information S6). Although increased perceived risk of future thyroid surgery impacted preferences for HT over AS and TT over HT, the effect was much smaller than that of increased cancer recurrence, without a clear association between those with benign nodules or decisional regret.

**FIGURE 4:**
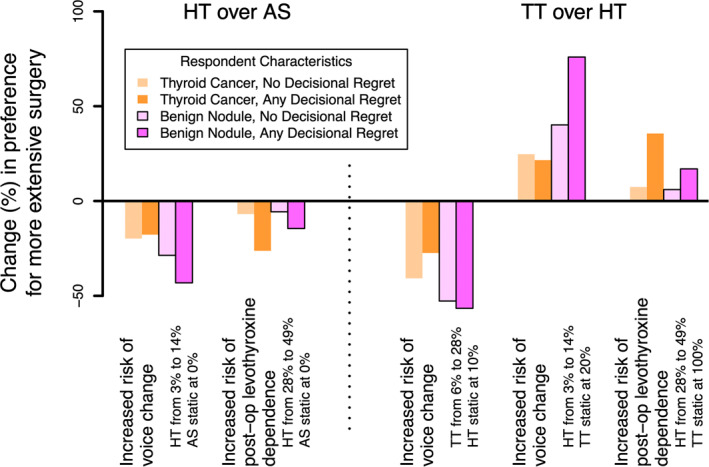
Choice preferences between HT and AS (panel A) and between TT and HT (panel B) following variation in perceived risks associated with surgery. For example, in Panel A, as the perceived risk of voice change increases from 3% to 14% following HT and remains static following AS, participants are less likely to choose HT over AS, with greatest change (∼40%) in participants with decisional regret and without a personal history of thyroid cancer. AS, active surveillance; HT, hemithyroidectomy; and TT, total thyroidectomy.

The effect of increased perceived risk of surgical complications on preferences is summarized in Figure 4. The perceived risk of permanent hypoparathyroidism had negligible effect on preferences and hence was not included in the graph. As the perceived risk of voice change after HT increased from 3% to 14%, more participants preferred AS. However, even where the perceived risk of voice change with TT was kept static at 20%, as the perceived risk of voice change with HT increased from 3% to 14% (still below the rate with TT), a strong preference toward TT was found, especially by those with benign nodules who expressed any decisional regret. The perceived risk of levothyroxine dependence altered surgical preferences predominantly for those who reported decisional regret with minimal influence on those with no regret regardless of their history of thyroid cancer. Equivalence analysis for voice change revealed that when the perceived risk of voice change associated with HT reached 2.2%, the preferences for TT and HT were equal (each preferred by 25% of respondents). However, at this level of perceived risk of voice change, 50% of participants preferred AS.

## 4 Discussion

To avoid overtreatment of low‐risk DTC, and overcome the risk of historical practice bias, robust evidence regarding patient preferences and values regarding treatment decisions are required. This study reports the outcome of a methodologically robust DCE around patient preferences regarding values informing initial treatment decisions. The key finding is that when typical clinical risks are applied, and when blinded to the actual treatment, patients prefer the perceived risks and experiences associated with AS rather than TT or HT. For those with any level of decisional regret following surgery, the preference for a less invasive approach was even stronger. Across all scenarios, the strongest attribute contributing to decisional choice was perceived risk of cancer recurrence.

### 4.1 Patient Preferences for Active Surveillance

These data demonstrate patients' preference for the decisional attributes associated with AS (49% in this study), which remains discordant with the low uptake of AS in clinical practice [39, 40]. Barriers to AS include clinician practice patterns and attitudes, established protocols and systems for patient follow‐up, and real or perceived patient barriers [11, 41]. Alternately, AS has also been shown to have a lower baseline of anxiety compared to patients undergoing immediate surgery [42]. Our findings suggest that patients' preferences for AS may not be adequately recognized in clinical practice [41]. Data from Japan, where AS follow‐up programs are well established, suggest high levels of patient retention and satisfaction with AS programs, particularly when undertaken in a collaborative and supportive clinician–patient relationship [39, 43].

### 4.2 Fear of Cancer Recurrence

Fear of cancer recurrence (FCR) is common in thyroid cancer survivors and often disproportionate to the true risk of recurrence [44]. Data from this study confirm that FCR is the strongest single attribute driving patient decisions, findings that are confirmed in other DCEs. Ahmadi et al. found that participants were willing to “trade‐off” some level of FCR to mitigate other harms, whereas Dixon et al. found that “labeling” thyroid cancer as either a lesion or nodule (rather than a cancer) altered participants' choice of management [19, 20]. In clinical practice, it is critical that surgeons acknowledge the powerful impact of the word “cancer” on decision‐making, as this can lead to more aggressive surgical intervention. Patients may need time to adjust their expectations to the reality of the lower risk profile of thyroid cancer before making well‐informed and balanced decisions [45].

### 4.3 Surgical Complications

Interestingly, the risk attributes associated with surgical complications were less significant in the patient decision than FCR. Of surgical complications, voice change was found to have the greatest impact on decisional choice rather than need for pharmacotherapy. The incidence of self‐reported voice change in the study cohort was high in this study and may relate in part to the timing of the survey. The wording for voice change in this DCE of “noticeable voice change” allowed patients to make subjective decisions for themselves about the extent of voice change rather than relying on validated voice measures or evidence of nerve dysfunction.

It is noteworthy that the perceived risk of requiring calcium supplements did not influence decision‐making in this study. Despite literature demonstrating significant patient burden and health related quality of life detriments with permanent hypoparathyroidism [46], it is likely that statements, such as “the need to take tablets to treat your calcium levels,” fail to communicate these wider impacts to patients. Other literature also suggests that calcium supplementation does not drive decisions about thyroid cancer [47]. The discord between the true quality of life implications of hypocalcaemia and the benign nature of the explanation is important for clinicians to understand when explaining risks to patients. This is but one example of how SDM should focus on meaningful quality of life outcomes contextualized for the patient rather than solely on traditional disease or surgical risks [14, 48].

### 4.4 Impact of Decisional Regret

Decisional regret can be associated with adverse physical outcomes that impair quality of life, increase patient anxiety, and lead to high levels of decisional conflict [49, 50]. In this study, all patients who had undergone thyroid surgery were asked to complete a decisional regret scale, with 60% reporting no regret regarding their surgical choice. For those reporting regret, voice changes drove regret for most. Decisional regret was measured by Sawka et al. as part of a trial of low‐risk thyroid cancer patients who underwent shared decision‐making and chose either AS or surgery (HT) [10]. Overall rates of decision regret were very low regardless of treatment decision, but higher decisional regret was recorded in patients who elected to cross‐over from AS to HT. There is emerging evidence in other cancer types that SDM decreases decisional regret, increases patient knowledge, and may lead to patients choosing less invasive surgical options [13]. There is a need to evaluate patient outcomes and decision satisfaction following SDM in thyroid cancer management.

### 4.5 Unlabeled Options

The presentation of unlabeled treatment options in this hypothetical scenario allows delineation between the attributes associated with a treatment choice and the treatment choice itself. The study vignette clearly introduced that the hypothetical patient had “cancer” and that the three options presented represented no surgery, HT, and TT. However, when each unlabeled option was presented in the scenario (as option A, B, and C), the order was randomized. The options could not easily be equated to the extent of surgery. Despite this, it remains possible that some patients did not appreciate that no surgery would be offered with one of the options (AS) and that the cancer would remain in situ. Nevertheless, omission of labels has a powerful impact on patient decision‐making and choice. In two prior DCEs examining the extent of treatment of thyroid cancer, when the word “cancer” was omitted [20, 22], patients chose a less invasive option. Although in clinical practice, the term cancer should not be avoided, care should be taken to communicate the typically excellent prognosis of a thyroid cancer diagnosis when presenting treatment options.

### 4.6 Limitations and Strengths

Although this DCE aimed to present a realistic scenario of low‐risk thyroid cancer to patients and elicit preferences, it remains hypothetical and may not replicate a participant's actual experience. We acknowledge that although the chosen five attributes and levels were set from the literature, other factors may be important for patient decision‐making. Although the use of unlabeled treatment options aimed to focus patients' attention on the trade‐offs of the attributes (without preconceived associations regarding the treatment options themselves), it is possible that patients' understanding of both attributes and treatment options may differ between this survey and the level of understanding obtained within a clinician–patient consultation, particularly if part of a formalized SDM process. It is also important to note that the clinical effect of hypocalcemia on a patient's quality of life was not explored and therefore, may underrepresent the effect that this could have on a patient's choice. The exclusion of patients awaiting surgery minimized any unintended consequences of the research on real‐life clinical decisions but also excluded the group for whom the research was most relevant. Clinical data regarding patients were not collected including indication for surgery, presence of compressive symptoms, thyroid function status, comorbidities, type or extent of malignancy, prior radioactive iodine, and prior health experiences. We acknowledge that these and other factors can influence decision‐making, treatment satisfaction, and survey participation. It is also possible that recall and selection bias may have influenced the results. Self‐selection bias should also be considered when interpreting the decisional regret influence. Participants who experience regret (40% in this study) or inversely are more satisfied may be more motivated to participate, skewing the responses. The cost of care was also not considered in this DCE. Within Australia's universal healthcare system, all patients have access to care but out of pocket expenses can vary. In this hypothetical scenario, it cannot be fully assumed, but is unlikely that costs of care contributed to preferences. Nevertheless, this study provides data from patients with thyroid nodular disease (benign and malignant), most of whom have undergone a thyroid operation, regarding the relative importance of clinical factors and trade‐offs that patients are willing to make when considering the extent of surgery for low‐risk DTC. The perspectives of these groups of patients are vital to informing current clinical practice and future clinical research.

## 5 Conclusion

In this hypothetical scenario, when presented with relative risks associated with blinded treatment options for small low‐risk DTC, most patients prefer active surveillance. When discussing treatment options with patients with small low‐risk DTC, clinicians should ensure accurate individualized communication of relative risks of both the cancer and treatment options. Where this is communicated with respect to an individual patients' educational level, cultural background, and prior experience, patients can be empowered to partake in shared decision‐making.

## Author Contributions

**Jacob Hampton:** conceptualization, investigation, methodology, project administration, resources, software, visualization, writing – original draft, writing – review and editing. **Gavin Cooper:** conceptualization, data curation, formal analysis, investigation, methodology, software, visualization. **Laura Wall:** conceptualization, data curation, formal analysis, Investigation, methodology, visualization, writing – original draft, writing – review and editing. **Christopher Rowe:** conceptualization, investigation, methodology, visualization, writing – original draft, writing – review and editing. **Nicholas Zdenkowski:** conceptualization, investigation, methodology, supervision, validation, visualization, writing – original draft, writing – review and editing. **Elizabeth Fradgley:** conceptualization, investigation, methodology, supervision, validation, visualization. **Julie Miller:** resources, visualization, writing – review and editing. **Jenny Gough:** resources, visualization, writing – review and editing. **Scott D Brown:** conceptualization, data curation, formal analysis, investigation, methodology, supervision, validation, visualization, writing – original draft, writing – review and editing. **Christine O'Neill:** conceptualization, investigation, methodology, project administration, resources, supervision, validation, Visualization, writing – original draft, writing – review and editing.

## Disclosure

The authors have nothing to report.

## Consent

Informed consent was obtained from all individual participants included in the study.

## Conflicts of Interest

The authors declare no conflicts of interest.

## Supporting information

Supplementary Information S1

Supplementary Information S2

Supplementary Information S3

Supplementary Information S4

Supplementary Information S5

Supplementary Information S6
