# Supplementary material for: Risk of Cancer Recurrence Exerts the Strongest Influence on Choice Between Active Surveillance and Thyroid Surgery as Initial Treatment for Low‐Risk Thyroid Cancer: Results of a Discrete Choice Experiment
Source: World J Surg. 2025 Mar 5;49(5):1254–63. doi: 10.1002/wjs.12520 (PMC12058448; doi:10.1002/wjs.12520)
Supplement: Supplementary file 1 — Supplementary Information S1 [file WJS-49-1254-s003.pdf]

Online Resource 1

Risk of cancer recurrence exerts the strongest influence on choice between active surveillance and thyroid surgery as initial treatment for low-risk thyroid cancer: results of a discrete choice experiment

World Journal of Surgery

Jacob Hampton, Gavin Cooper, Laura Wall, Christopher Rowe, Nicholas Zdenkowski, Elizabeth Fradgley, Julie Miller, Jenny Gough, Scott Brown, Christine O’Neill

Corresponding Author:

Conjoint Associate Professor Christine J O’Neill[1-3]

Surgical Services, John Hunter Hospital

Locked Bag 1, Hunter Regional Mail Centre

Newcastle NSW, 2310, Australia

christine.oneill@newcastle.edu.au

[1] Surgical Services John Hunter Hospital, Newcastle NSW Australia
[2] School of Medicine and Public Health, University of Newcastle, Newcastle NSW Australia
[3] Hunter Medical Research Institute, Newcastle NSW Australia

Online Resource 1

Participant resource –
Survey postcard given to patients for recruitment.

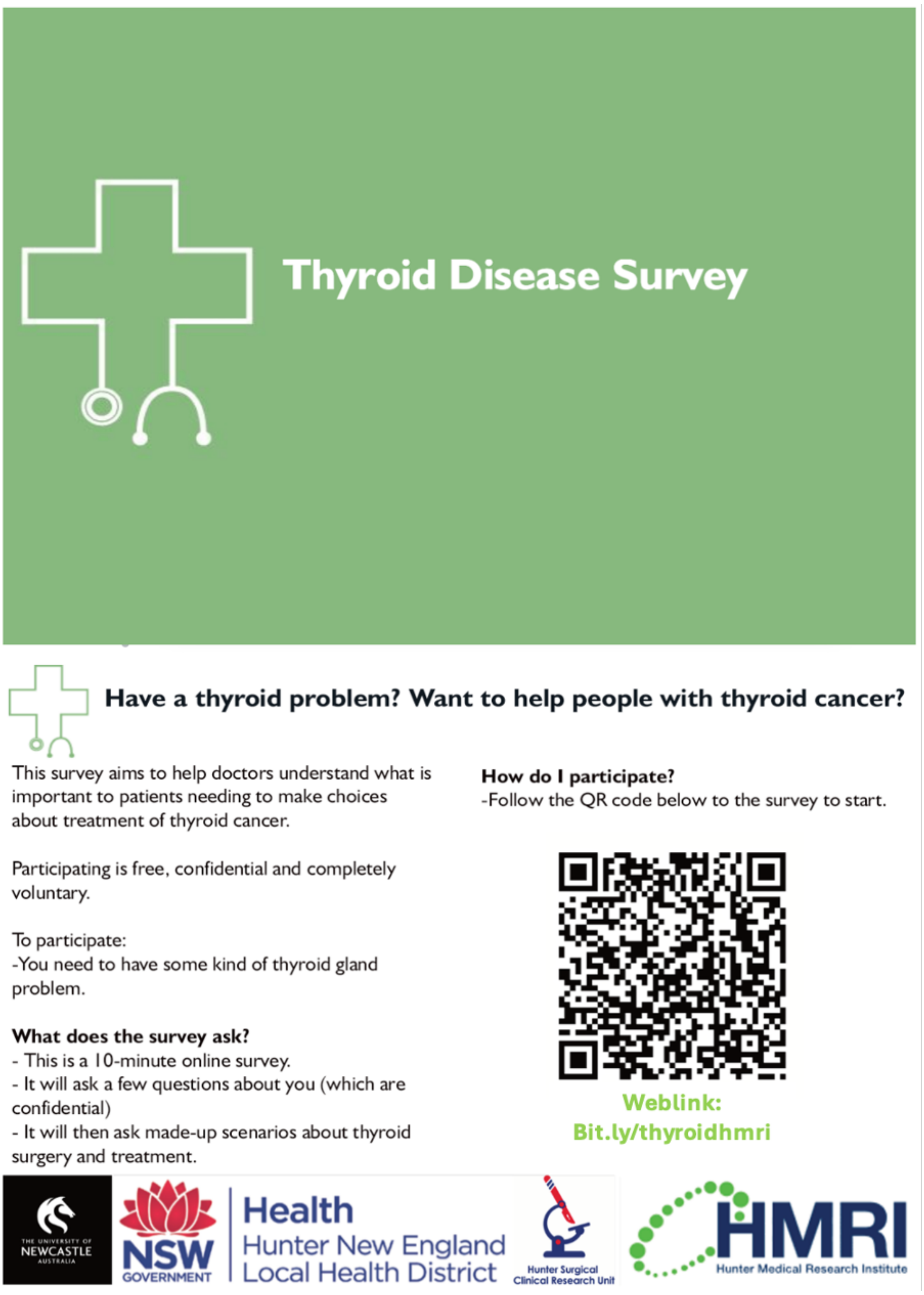

Clinician Resource -
The following is an example of the recruiting card given to the recruiting clinicians. The aim was to ensure they has easy access to the inclusion and exclusion criteria do reduce the risk of an ineligible patient being recruited for the survey.

Thyroid discrete choice survey recruitment

Inclusion criteria:

- Age $\geq$ 18 years
- Have a diagnosis of thyroid nodules that:
  - Have been clinically assessed as being benign,
    OR
  - ATA low risk malignant
- Either
  - Already had surgery for a thyroid nodule
    OR
  - Not recommended to undergo surgery for their thyroid nodule

Exclusion criteria:

- ATA Intermediate or high-risk malignant thyroid cancers
- Pre-operative patients for thyroid surgery
