# Supplementary material for: Risk of Cancer Recurrence Exerts the Strongest Influence on Choice Between Active Surveillance and Thyroid Surgery as Initial Treatment for Low‐Risk Thyroid Cancer: Results of a Discrete Choice Experiment
Source: World J Surg. 2025 Mar 5;49(5):1254–63. doi: 10.1002/wjs.12520 (PMC12058448; doi:10.1002/wjs.12520)

Online Resource 2

Risk of cancer recurrence exerts the strongest influence on choice between active surveillance and thyroid surgery as initial treatment for low-risk thyroid cancer: results of a discrete choice experiment

World Journal of Surgery

Jacob Hampton, Gavin Cooper, Laura Wall, Christopher Rowe, Nicholas Zdenkowski, Elizabeth Fradgley, Julie Miller, Jenny Gough, Scott Brown, Christine O'Neill

Corresponding Author:

Conjoint Associate Professor Christine J O'Neill[1-3]

Surgical Services, John Hunter Hospital

Locked Bag 1, Hunter Regional Mail Centre

Newcastle NSW, 2310, Australia

christine.oneill@newcastle.edu.au

[1] Surgical Services John Hunter Hospital, Newcastle NSW Australia
[2] School of Medicine and Public Health, University of Newcastle, Newcastle NSW Australia
[3] Hunter Medical Research Institute, Newcastle NSW Australia

Online Resource 2

Information about the “Show Explanation” button.

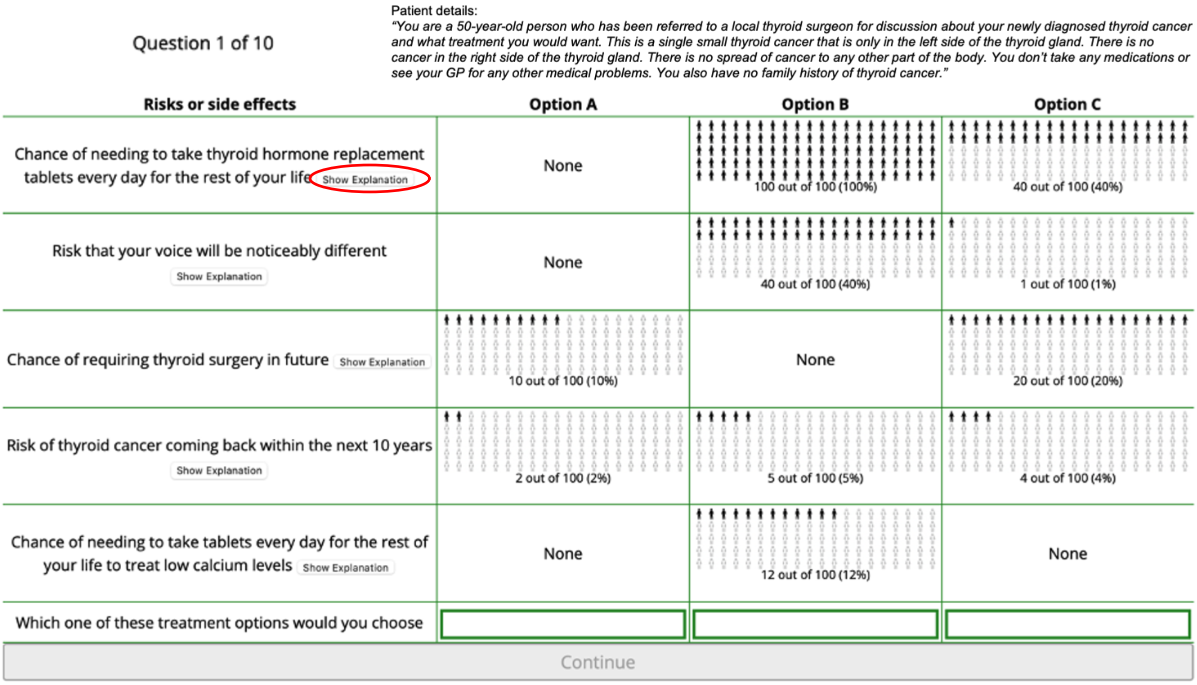

Question 1 of 10

Patient details:
“You are a 50-year-old person who has been referred to a local thyroid surgeon for discussion about your newly diagnosed thyroid cancer and what treatment you would want. This is a single small thyroid cancer that is only in the left side of the thyroid gland. There is no cancer in the right side of the thyroid gland. There is no spread of cancer to any other part of the body. You don’t take any medications or see your GP for any other medical problems. You also have no family history of thyroid cancer.”

Risks or side effects	Option A	Option B	Option C
Chance of needing to take thyroid hormone replacement tablets every day for the rest of your life Show Explanation	None	100 out of 100 (100%)	40 out of 100 (40%)
Risk that your voice will be noticeably different Show Explanation	None	40 out of 100 (40%)	1 out of 100 (1%)
Chance of requiring thyroid surgery in future Show Explanation	10 out of 100 (10%)	None	20 out of 100 (20%)
Risk of thyroid cancer coming back within the next 10 years Show Explanation	2 out of 100 (2%)	5 out of 100 (5%)	4 out of 100 (4%)
Chance of needing to take tablets every day for the rest of your life to treat low calcium levels Show Explanation	None	12 out of 100 (12%)	None
Which one of these treatment options would you choose			

Continue

The “show explanation” button was developed to expand the participants knowledge around the attribute to assist them with answering the question.

This information was presented during the DCE component of the survey but only if patients needed to read it to assist with answering the question. Next to each of the five risks or side effects, there was a “Show Explanation” button which can be seen above. By clicking this button, a more detailed explanation would appear to assist with answering the question. Please find here the content available when clicking the button:

Risk that your voice will be noticeably different. This may be due to a slight change in the character of your voice due to thyroid tissue being removed from near the voice box, or occasionally due to damage to nerves that control your voice box during the operation. .

Risk of thyroid cancer coming back within the next 10 years. If thyroid cancer comes back, it most often comes back as a small lump in the neck, which can be removed with an operation, and generally still has a good prognosis.

Chance of needing to take thyroid hormone replacement tablets for the rest of your life. The tablet contains thyroid hormone, that is the same as your body can produce and needs to be taken every day for the rest of your life. There are no side effects if the dose is correct.

Chance of needing to take tablets every day for the rest of your life to treat low calcium levels. These tablets are generally tolerated well but must be taken every day to prevent symptoms of low calcium.

Chance of requiring thyroid surgery in the future, which would be recommended if the cancer was found to be more advanced than expected and you needed more treatment.

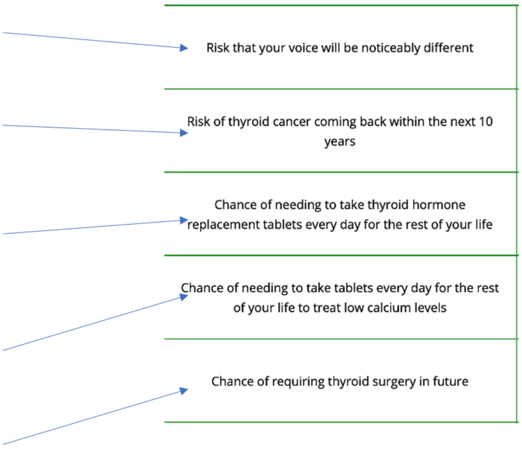

Supplement: Supplementary file 2 — Supplementary Information S2 [file WJS-49-1254-s004.pdf]
