# Supplementary material for: Risk of Cancer Recurrence Exerts the Strongest Influence on Choice Between Active Surveillance and Thyroid Surgery as Initial Treatment for Low‐Risk Thyroid Cancer: Results of a Discrete Choice Experiment
Source: World J Surg. 2025 Mar 5;49(5):1254–63. doi: 10.1002/wjs.12520 (PMC12058448; doi:10.1002/wjs.12520)
Supplement: Supplementary file 3 — Supplementary Information S3 [file WJS-49-1254-s006.pdf]

Online Resource 3

Risk of cancer recurrence exerts the strongest influence on choice between active surveillance and thyroid surgery as initial treatment for low-risk thyroid cancer: results of a discrete choice experiment

World Journal of Surgery

Jacob Hampton, Gavin Cooper, Laura Wall, Christopher Rowe, Nicholas Zdenkowski, Elizabeth Fradgley, Julie Miller, Jenny Gough, Scott Brown, Christine O'Neill

Corresponding Author:

Conjoint Associate Professor Christine J O'Neill[1-3]

Surgical Services, John Hunter Hospital

Locked Bag 1, Hunter Regional Mail Centre

Newcastle NSW, 2310, Australia

christine.oneill@newcastle.edu.au

[1] Surgical Services John Hunter Hospital, Newcastle NSW Australia
[2] School of Medicine and Public Health, University of Newcastle, Newcastle NSW Australia
[3] Hunter Medical Research Institute, Newcastle NSW Australia

Online Resource 3

Example of the online survey (excluding the Discrete Choice Experiment which is seen in Figure 1)

Please complete the survey below.

What is your current age?	◯ 18 to 29<br>◯ 30 to 39<br>◯ 40 to 49<br>◯ 50 to 59<br>◯ 60 to 69<br>◯ 70+
What is your gender?	◯ Female<br>◯ Male<br>◯ Other<br>◯ Neither or prefer not to say
Are you of Aboriginal or Torres Strait Islander origin?	◯ Neither<br>◯ Yes, Aboriginal<br>◯ Yes, Torres Strait Islander<br>◯ Both Aboriginal and Torres Strait Islander<br>◯ Prefer not to say
If you would like the research team to arrange for an Aboriginal Liaison Officer to contact you to support you through this survey, you can speak to a member of the research team by contacting us on:<br><br>- Email: HNELHD-SurgeryResearch@health.nsw.gov.au<br>or<br>- Phone 02 4923 6397	
What is your residential postcode?	______________________
How did you find out about this study?	◯ Phone call from an investigator<br>◯ Postcard from your Doctor
Where did you see a specialist for your thyroid gland problem?	◯ Hunter Region<br>◯ Port Macquarie<br>◯ Sydney<br>◯ Melbourne<br>◯ Brisbane<br>◯ Other
Please specify where?	______________________
What kind of thyroid gland problem do you have?	◯ Cancer<br>◯ Non-cancer thyroid problem<br>◯ Unsure
How many thyroid operations have you had?	◯ no operation<br>◯ 1 operation<br>◯ 2 operations<br>◯ 3+ operations
What date was your most recent operation?<br><br>**Note** You do not need an exact date. For example: if you think you operation was in March 2019, just put in 01/03/2019	______________________

Do you need to take calcium tablets?	◯ Yes<br>◯ No
Are you on calcium tablets as a result of your thyroid surgery?	◯ Yes<br>◯ No
Compared to prior to your operation, does your voice sound different or not as strong?	◯ Yes<br>◯ No
Do you now take thyroid hormone tablets?	◯ Yes<br>◯ No

Please answer each of the 5 questions below.

In your personal experience with thyroid disease, do you regret your decision to have or not have an operation on your thyroid gland?

	Strongly Agree	Agree	Neither Agree Nor Disagree	Disagree	Strongly Disagree
It was the right decision	◯	◯	◯	◯	◯
I regret the choice that was made	◯	◯	◯	◯	◯
I would go for the same choice if I had to do it over again	◯	◯	◯	◯	◯
The choice did me a lot of harm	◯	◯	◯	◯	◯
The decision was a wise one	◯	◯	◯	◯	◯

Please answer the final questions for the survey below.

Thank you greatly for your time!

We have just asked you questions based on a made-up scenario.<br>If you were the patient in this scenario, Would you like additional information beyond your consultation with your doctor about treatment options?	◯ Yes<br>◯ No<br>◯ Not sure
Would you like this information provided to you online or in a written format?	◯ Online<br>◯ Written<br>◯ Both
If you decided you did want more information, would you like this provided to you online or in a written format?	◯ Online<br>◯ Written<br>◯ Both<br>◯ Not sure

Thank you for taking the time to complete this survey. It is greatly appreciated. If you have any concerns and would like to discuss this project with someone you can speak to a member of the research team by contacting the Research Team, via email: HNELHD-SurgeryResearch@health.nsw.gov.au or phone 02 4923 6397.

If participation in this study causes any distress, you should contact someone for assistance or support. People or services available to assist that you may wish to contact are:

- A trusted friend or family member
- Your local doctor (GP)
- The research team - with the contacts above
- Lifeline Australia - P: 13 11 14
- Cancer Council New South Wales - P: 13 11 20

You may close the browser at any time.

Decisional regret scale calculation.

The Decision Regret Scale is a validated, 5-item scale with scores from 0–100. A five-point Likert scale is used to assess these and range from 1 = strongly agree to 5 = strongly disagree. The mean scores are obtained and then converted by subtracting 1 and multiplying by 25. A score of 0 is considered no regret, 1–24 is mild regret and greater than or equal to 25 is severe regret.
Items 2 and 4 are reverse coded so that, for each item, a higher number will indicate more regret. Therefore, in the thyroid example used above the items scores for "I regret the choice that I made" and "the choice did me a lot of harm" were reverse coded to obtain the mean score. The mean scores were than used for the purpose of this research to define between no regret, mild regret and severe regret.

The resource used to complete a decisional regret scale can be found here:

https://decisionaid.ohri.ca/docs/develop/User_manuals/UM_Regret_Scale.pdf
