# Supplementary material for: Risk of Cancer Recurrence Exerts the Strongest Influence on Choice Between Active Surveillance and Thyroid Surgery as Initial Treatment for Low‐Risk Thyroid Cancer: Results of a Discrete Choice Experiment
Source: World J Surg. 2025 Mar 5;49(5):1254–63. doi: 10.1002/wjs.12520 (PMC12058448; doi:10.1002/wjs.12520)
Supplement: Supplementary file 4 — Supplementary Information S4 [file WJS-49-1254-s005.pdf]

Online Resource 4

Risk of cancer recurrence exerts the strongest influence on choice between active surveillance and thyroid surgery as initial treatment for low-risk thyroid cancer: results of a discrete choice experiment

World Journal of Surgery

Jacob Hampton, Gavin Cooper, Laura Wall, Christopher Rowe, Nicholas Zdenkowski, Elizabeth Fradgley, Julie Miller, Jenny Gough, Scott Brown, Christine O'Neill

Corresponding Author:

Conjoint Associate Professor Christine J O'Neill[1-3]

Surgical Services, John Hunter Hospital

Locked Bag 1, Hunter Regional Mail Centre

Newcastle NSW, 2310, Australia

christine.oneill@newcastle.edu.au

[1] Surgical Services John Hunter Hospital, Newcastle NSW Australia
[2] School of Medicine and Public Health, University of Newcastle, Newcastle NSW Australia
[3] Hunter Medical Research Institute, Newcastle NSW Australia

Online Resource 4

Decision regret scale sub-analysis

Decisional regret			
	None	Mild	Severe
N= 132	79 (60%)	36 (27%)	17 (13%)
Low-risk DTC	33 (42%)	16 (44%)	6 (35%)
Benign nodule	46 (58%)	20 (56%)	11 (65%)
Calcium	7 (9%)	4 (11%)	1 (6%)
Voice change	14 (18%)	14 (39%)	6 (35%)
Thyroxine	31 (39%)	14 (39%)	10 (59%)

Calcium: New calcium supplementation following thyroid treatment
Voice change: New voice change following thyroid treatment
Thyroxine: New thyroid hormone replacement following thyroid treatment
