# Supplementary material for: Risk of Cancer Recurrence Exerts the Strongest Influence on Choice Between Active Surveillance and Thyroid Surgery as Initial Treatment for Low‐Risk Thyroid Cancer: Results of a Discrete Choice Experiment
Source: World J Surg. 2025 Mar 5;49(5):1254–63. doi: 10.1002/wjs.12520 (PMC12058448; doi:10.1002/wjs.12520)
Supplement: Supplementary file 5 — Supplementary Information S5 [file WJS-49-1254-s001.pdf]

Online Resource 5

Risk of cancer recurrence exerts the strongest influence on choice between active surveillance and thyroid surgery as initial treatment for low-risk thyroid cancer: results of a discrete choice experiment

World Journal of Surgery

Jacob Hampton, Gavin Cooper, Laura Wall, Christopher Rowe, Nicholas Zdenkowski, Elizabeth Fradgley, Julie Miller, Jenny Gough, Scott Brown, Christine O'Neill

Corresponding Author:

Conjoint Associate Professor Christine J O'Neill[1-3]

Surgical Services, John Hunter Hospital

Locked Bag 1, Hunter Regional Mail Centre

Newcastle NSW, 2310, Australia

christine.oneill@newcastle.edu.au

[1] Surgical Services John Hunter Hospital, Newcastle NSW Australia
[2] School of Medicine and Public Health, University of Newcastle, Newcastle NSW Australia
[3] Hunter Medical Research Institute, Newcastle NSW Australia

Online Resource 5

Conditional logit Model (CLM)

Attribute	Coefficient (β)	SE (of β)	Z	*p*	Exponentiated coefficient (odds ratio)	CI (lower 95%)	CI (lower 95%)
Chance of needing to take tablets every day for the rest of your life to treat low calcium levels	3.102	0.739	4.198	<0.001	22.250	5.228	94.70
Risk that your voice will be noticeably different	-2.846	0.337	-8.441	<0.001	0.058	0.030	0.113
Chance of needing to take thyroid hormone replacement tablets every day for the rest of your life	-1.136	0.233	-4.870	<0.001	0.321	0.203	0.507
Chance of requiring thyroid surgery in future	-2.301	0.512	-4.492	<0.001	0.100	0.037	0.273
Risk of thyroid cancer coming back within the next 10 years	-9.876	0.684	-14.437	<0.001	5.140^{e-05}	1.345^{e-05}	1.964^{e-04}

Coefficients and their standard error (SE), z score, p value and the exponentiated coefficients (odds ratios) of those coefficients and the 95% confidence interval for those odds ratios, from the conditional logit model with all attributes included as main effects, stratified by choice set.
