# Supplementary material for: Risk of Cancer Recurrence Exerts the Strongest Influence on Choice Between Active Surveillance and Thyroid Surgery as Initial Treatment for Low‐Risk Thyroid Cancer: Results of a Discrete Choice Experiment
Source: World J Surg. 2025 Mar 5;49(5):1254–63. doi: 10.1002/wjs.12520 (PMC12058448; doi:10.1002/wjs.12520)
Supplement: Supplementary file 6 — Supplementary Information S6 [file WJS-49-1254-s002.pdf]

Online Resource 6

Risk of cancer recurrence exerts the strongest influence on choice between active surveillance and thyroid surgery as initial treatment for low-risk thyroid cancer: results of a discrete choice experiment

World Journal of Surgery

Jacob Hampton, Gavin Cooper, Laura Wall, Christopher Rowe, Nicholas Zdenkowski, Elizabeth Fradgley, Julie Miller, Jenny Gough, Scott Brown, Christine O'Neill

Corresponding Author:

Conjoint Associate Professor Christine J O'Neill[1-3]

Surgical Services, John Hunter Hospital

Locked Bag 1, Hunter Regional Mail Centre

Newcastle NSW, 2310, Australia

christine.oneill@newcastle.edu.au

[1] Surgical Services John Hunter Hospital, Newcastle NSW Australia
[2] School of Medicine and Public Health, University of Newcastle, Newcastle NSW Australia
[3] Hunter Medical Research Institute, Newcastle NSW Australia

Online Resource 6

How treatment options change when there is a change to risk of cancer recurrence over all participants.

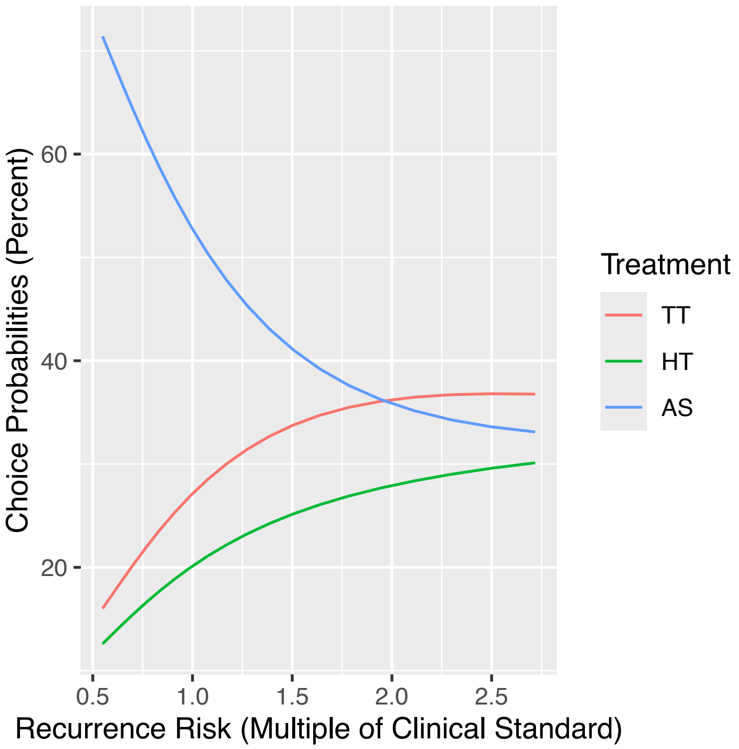

TT – Total thyroidectomy, HT – Hemithyroidectomy, AS – Active surveillance.

This figure shows how the choice preference between the different treatment options (red - TT, green - HT, and blue lines – AS) can change in response to changes in the risk of cancer recurrence relative to clinical standard risks (x-axis). Points greater than 1.0 on the x-axis (i.e. to the right) show that when risk increases, the probability of choosing active surveillance decreases and the probability of choosing a surgery (either TT or HT) increases. When the risk of recurrence is almost double clinical standard (x=2.0), choices for total thyroidectomy exceed choices for active surveillance. Points smaller than 1.0 on the x-axis (i.e. to the left) show that when risk decreases relative to clinical standard, active surveillance is even more strongly preferred over surgical options.

How treatment options change when there is a change to risk of cancer recurrence in four subgroups of participants.

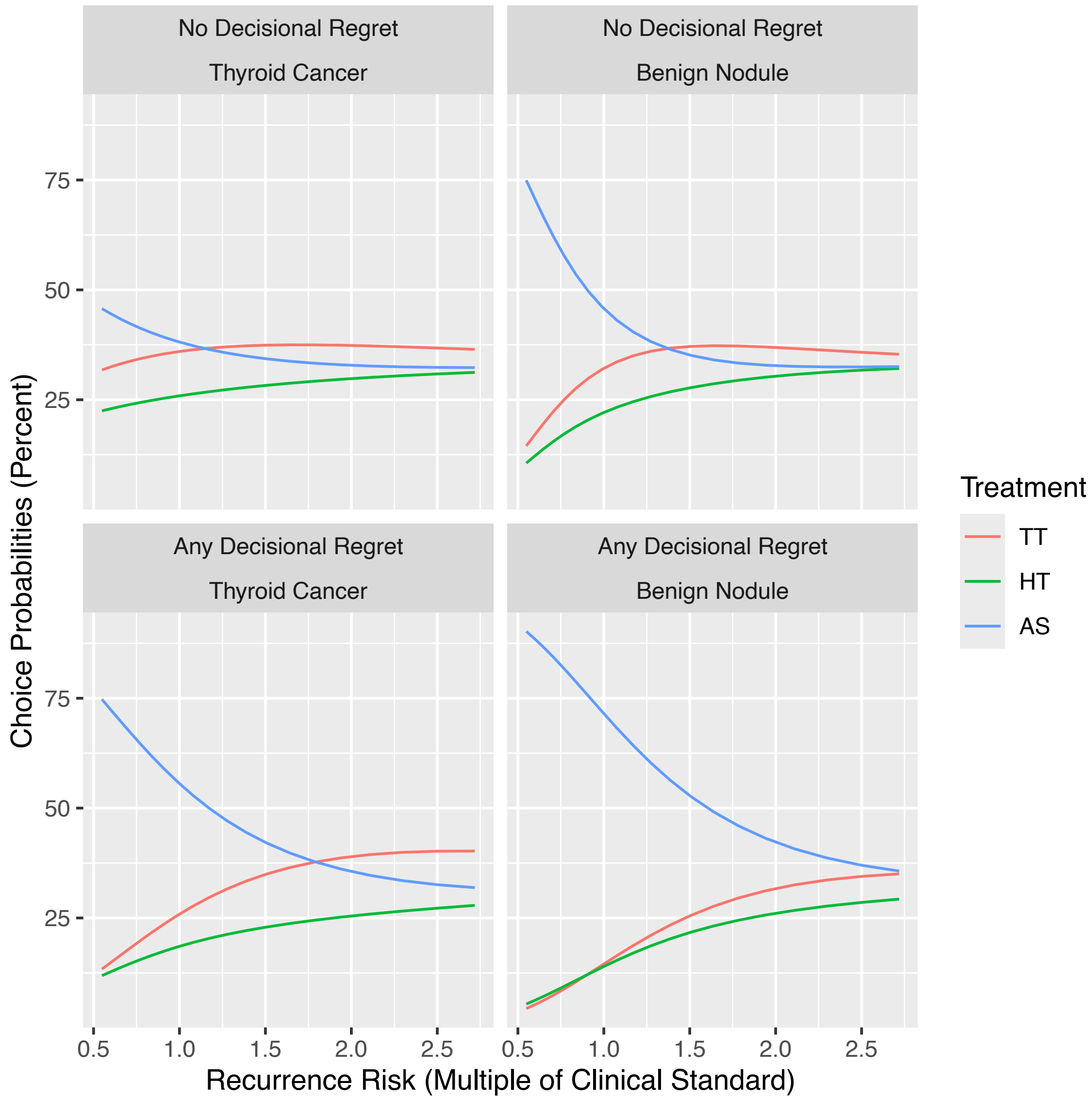

TT – Total thyroidectomy, HT – Hemithyroidectomy, AS – Active surveillance.

For this analysis, the only changes were that participants were stratified into four groups. This was according to whether they had thyroid cancer (left column) or a benign nodule (right column) and whether they had no decisional regret (top row) or any decisional regret (bottom row). Differences between strata show that lived experience with thyroid cancer and decisional regret both made people more likely to preference surgery over AS compared to those with benign nodules and no regret.
